# Modern Sociology

**Published:** 1902-11-22

**Authors:** 


### Modern Sociology.

### BOY AND GIRL CLERKS.

Some of the London omnibuses are just now displaying among advertisements of the theatres the legend "Learn to Earn." It is intended to appeal to that large class of boys and girls who have ambitions, and to whom the prospect of an office stool, a weekly salary and "your evenings free" is an alluring one. Formerly many of these same young people would have sought employment in humbler and more restricted spheres of life; or, in some cases—this applies to the girls, of course—would have idled away their time at home, in tricking themselves out in the hope of attracting offers of marriage. Ambition, *pace* Cardinal Wolsey, is undoubtedly a very good thing, and few of us would make much headway without it, but when one sees hundreds of these young people at our large technical training schools, filling room after room where shorthand, typewriting, modern languages and other subjects necessary to a fully-equipped clerk are being busily taught day by day, one cannot help a feeling of depression at the sight. "What is to become of them all?" one asks involuntarily.

Might not many of them lead a healthier and happier life in the country, even—though no doubt they would scorn the suggestion—as domestic servants than they will live as clerks? One may see them any day in the restaurants; many of them are pale and unhealthy looking, spiritless and jaded. The pity of it! Sad stories are told of girl clerks who marry without the smallest knowledge of domestic economy or the duties of mothers; but of course the permanent spinster has also to be taken into consideration; and since the demand for lady clerks appears to be increasing, it is useless to cry out against it.

From this point of view, then, it is encouraging to learn that although more than ever is expected in the way of qualifications, the prospects of employment are as bright as ever. For girls, there appears to be an increase of better-class positions; though more is demanded, the pay is better, and as many girls of good education are entering the ranks of lady clerks and secretaries, these high demands can be met. The best salaries are paid by large business houses, and positions in them are more secure than, for example, that of secretary to an author or other professional man. Whatever the reason, there appear to be more openings for youths than there are applicants to fill them at the present moment. The majority of the boys in training at Pitman's School are the sons of business men, preparing for entering their fathers' offices, and the manager of the school, part of whose work consists in maintaining a "Situation Bureau" for qualified students, has to refuse appointments on account of this "dearth of juniors." Upwards of a thousand vacancies are annually registered by the Bureau, which has been at work for nearly fifteen years, and all these are for appointments requiring some special skill or knowledge, such as can be acquired by undergoing a course of training in the school.

It is of course obvious to the most casual observer that enormous changes have taken place in business methods since the days when Newman Noggs snapped his fingers and Scrooge growled at the idle "clerk in the tank." The modern representative of Noggs has little leisure for snapping his fingers, unless he wishes to see his post snapped up before his eyes, and to find himself out of work. And the modern Scrooge, though he may yet find time to growl, is fully occupied in dictating his answers to correspondents, perhaps by means of the phonograph, while the millionaire members of a certain American firm are said to be so mindful of the value of time that they have had a special car on their morning train fitted up as an office, in which, during their hour's journey to New York, they open their morning's letters and dictate their replies and instructions without fear of disturbance. Noggs, with his quill pen, would be a little out of the running nowadays.

"The greatest changes in business methods," says a writer on the subject, "have been in the adaptation of tools, not merely for turning things out well, but for turning them out rapidly. In the one element of writing alone, it is a long way from the quill-pen office clerk to the typewriter of to-day. All along the line—steam, electricity, telegrams, telephones, and typewriters—men have been searching in twenty ways to find the shortest, and having found it there came changes of method in the use of the tools and appliances. In the economy of time and the classification of labour and of increased output, the plan of method in business has now a new meaning. It is not only order and neatness, but dispatch and rapidity and duplication of work. If a worker is to fit in with the new machinery, he must be trained to work in obedience to the law of the machine, and so, beyond the old qualification of the "three R's," office clerks have had to learn special methods of work. For a large modern business house is so different from the old counting-house of a past generation that the youth fresh from school, with his three R's, is like a hand weaver placed in the midst of the whirling looms of a cotton mill. Some old firms still prefer to train their juniors from the beginning, but generally, business men now expect a better preparation before entering the office than was ever known before, in some of those elements which hitherto have never found a place in ordinary schools; hence our special schools of business training to meet the demand. Certain things which form the alphabet, so to speak, of office work, have come to be recognised as things to be acquired before entering an office, both for the sake of the youth himself and for the convenience of the business man, who has no time to teach what he now expects should be inculcated in the outside education.

He goes on: "The great and overwhelming characteristic of modern methods is that of getting things done quickly, and shorthand and typewriting are indispensable to this end. But there is a danger of falling a little too much under this element of speed, of mere mechanism, and of setting up an automatic tyranny which has no consideration for nerves, and the possibility of physical breakdown in the workers. It has been represented to the writer by experienced typists that, where good-class work is concerned, there is a danger in this craving for speed of looking only to this one qualification, and to the amount of work that can be turned out by the worker as a part of the office machine. The fact that a lady typist is an expert and can run off letters at a rapid rate is too often considered a sufficient warrant for putting upon her a prolonged strain of work at high pressure, almost regardless of the fact of such physical strain. It is, of course, creditable to the typist that she has acquired the faculty of using the writing machine at the rate of 60 to 80 words a minute, but it is quite unfair to her to make that ability the excuse for employing insufficient hands to get through the day's correspondence, thus giving her no respite from morning to night, and obliging her sometimes to work late to clear off the heavy mass of work entrusted to her."

This, of course, constitutes a real danger, and it is one against which unions of typists and shorthand clerks will have to be on their guard.

